# Epicardial Adipose Tissue: A Multimodal Imaging Diagnostic Perspective

**DOI:** 10.3390/medicina61060961

**Published:** 2025-05-23

**Authors:** Giancarlo Trimarchi, Maria Ludovica Carerj, Concetta Zito, Gianluca Di Bella, Giovanni Taverna, Maurizio Cusmà Piccione, Pasquale Crea, Stefania Lo Giudice, Angela Buonpane, Michela Bonanni, Davide Restelli, Umberto Paradossi, Angelo Monteleone, Antonio Micari, Scipione Carerj

**Affiliations:** 1 Interdisciplinary Center for Health Sciences, Scuola Superiore Sant’Anna, 56127 Pisa, Italy; giancarlo.trimarchi18@gmail.com; 2 Fondazione Toscana G. Monasterio, Ospedale del Cuore G. Pasquinucci, 54100 Massa, Italy; michelabonanni91@gmail.com (M.B.); uparadossi@ftgm.it (U.P.); monteleone@ftgm.it (A.M.); 3 Centro Cardiologico Monzino IRCCS, 20138 Milan, Italy; 4 Department of Clinical and Experimental Medicine, University of Messina, 98100 Messina, Italy; czito@unime.it (C.Z.); gianluca.dibella@unime.it (G.D.B.); giovannitaverna95@gmail.com (G.T.); maurizio.cusmapiccione@polime.it (M.C.P.); pasqualecrea85@gmail.com (P.C.); stefania.logiudice1996@gmail.com (S.L.G.); 5 Department of Cardiovascular Sciences, Fondazione Policlinico Universitario A. Gemelli IRCCS, Università Cattolica Sacro Cuore, Largo Agostino Gemelli, 1, 00168 Roma, Italy; buonpaneangela@gmail.com; 6 Department of Experimental Medicine, University of Rome Tor Vergata, 00133 Rome, Italy; 7 Department of Cardio-Thoraco-Vascular Care, Azienda Socio Sanitaria Territoriale Lecco-Ospedale A. Manzoni, 23900 Lecco, Italy; dv.restelli@gmail.com; 8 Department of Biomedical and Dental Sciences and of Morphological and Functional Images, University of Messina, 98100 Messina, Italy; micariantonio@gmail.com

**Keywords:** epicardial adipose tissue, multimodality imaging, pericoronary adipose tissue, echocardiography

## Abstract

Epicardial adipose tissue (EAT), strategically located between the myocardium and the visceral pericardial layer, is increasingly recognized as an active player in cardiovascular health rather than a passive fat depot. EAT secretes a notable array of bioactive molecules known as adipokines, which exert critical exocrine and paracrine effects. Recent research has focused on pericoronary adipose tissue (PCAT)—the EAT surrounding coronary arteries—demonstrating its intricate bidirectional relationship with the vascular wall. Under normal physiological conditions, this interaction promotes vascular homeostasis; however, dysfunctional PCAT can release pro-inflammatory adipokines implicated in the pathogenesis of atherogenesis. Notably, PCAT inflammation has emerged as a significant factor associated with the development of coronary artery disease (CAD) and major cardiovascular events. This review seeks to elucidate the imaging methodologies employed to evaluate EAT, emphasizing cardiac computed tomography (CCT) as the preeminent imaging modality. Unlike echocardiography and cardiac magnetic resonance imaging, CCT not only visualizes and quantifies EAT but also concurrently assesses coronary arteries and PCAT. Recent findings have established the potential of CCT-derived PCAT attenuation as a noninvasive biomarker for coronary inflammation, offering prospects for monitoring therapeutic responses to innovative anti-inflammatory interventions in CAD management.

## 1. Introduction

In recent years, the visceral adipose tissue situated around the heart and coronary arteries, commonly referred to as epicardial adipose tissue (EAT), has garnered increasing attention. Accumulating evidence suggests that EAT may play a significant role in the development of obstructive coronary artery disease and the onset of acute coronary syndromes [1,2,3]. This emerging understanding positions EAT not merely as an inert substance but as a dynamic tissue that may influence cardiovascular health.

In the pursuit of enhancing cardiovascular risk assessment, noninvasive imaging techniques have increasingly been employed to evaluate and characterize EAT [4,5,6,7].

In this narrative review, we aim to provide a comprehensive understanding of EAT and pericoronary adipose tissue (PCAT) by first examining their anatomy and physiology under healthy conditions. We will explore how these adipose tissues function and interact within the body. Following this foundational overview, we will investigate the connection between EAT, PCAT, and the development of atherosclerosis, shedding light on how these tissues may contribute to or interact with the disease process. Finally, we will take a detailed look at the various imaging techniques used to visualize adipose tissue, with a particular emphasis on the role of cardiac computed tomography angiography (CCTA). Through this, we seek to highlight how CCTA is utilized in assessing PCAT, helping to improve our understanding and potentially guiding clinical practices related to cardiovascular health.

## 2. Anatomy and Distribution of Epicardial Adipose Tissue

EAT is defined as adipose tissue situated between the myocardium and the visceral layer of the pericardium, without any intervening fascial plane. A particularly notable subset of EAT is known as pericoronary adipose tissue (PCAT), which specifically encompasses the coronary arteries [8]. PCAT is characterized by the adipose tissue present within a radial distance from the outer vessel wall equivalent to the diameter of the adjacent coronary vessel [9]. PCAT is contiguous with the adventitial layer, while in smaller vessels and microvessels, the adipocytes in perivascular adipose tissue (PVAT) become an integral component of the vascular wall itself [10]. The second type, pericardial adipose tissue (PAT), is positioned between the visceral and parietal layers of the pericardium. This organization emphasizes the compartmentalization of adipose layers surrounding the heart, which may contribute to various physiological and pathological processes [11]. The third type is paracardial adipose tissue, which is located externally to the parietal pericardium, further contributing to the complexity of cardiac adipose distribution [12]. In a healthy heart, epicardial adipose tissue contributes significantly to cardiac anatomy by covering approximately 80% of the heart’s surface [13]. Importantly, the distribution of this fat is not uniform; it tends to concentrate more in regions such as the atrioventricular and interventricular grooves and predominantly around the epicardial coronary arteries. Conversely, smaller amounts of EAT can be detected around the atria, along the free wall of the right ventricle, and at the apex of the left ventricle. Histologically, EAT is composed of several distinct components, emerging from the splanchnopleuric mesoderm [14]. Notably, the adipocytes found in EAT are considerably smaller compared to those in peritoneal and subcutaneous fat [15]. In terms of its phenotypic characteristics, EAT manifests as brown adipose tissue during early developmental stages, transitioning to exhibit features of “beige” fat during middle age—an amalgamation of white and brown adipocytes [16]. Additionally, EAT’s microenvironment is enriched with inflammatory, stromal, and immune cells, complemented by nervous and nodal tissues [17]. This intricate composition underscores the multifaceted role of epicardial adipose tissue not only in energy storage but also in immunological and cardiovascular functions.

## 3. Physiology of EAT

EAT plays a multifaceted role in maintaining cardiovascular health. EAT has been acknowledged as a safeguarding layer for the heart, particularly in providing protection to the coronary arteries against the torsional effects elicited by arterial pulse waves and cardiac contraction [18]. In addition to its structural role, the epicardial adipose tissue serves as a protective barrier against pathogens, enveloping essential cardiac structures with immune activity [18]. EAT comprises adipocytes that display either brown or beige characteristics, which are crucial for thermogenesis in response to cold stimuli. This thermogenic capability plays a vital role in protecting the heart from hypothermia [19]. Moreover, EAT is characterized by its significant lipolytic and lipogenic metabolic activities. This position allows EAT to serve as an immediate source of energy for the myocardium by swiftly releasing fatty acids during periods of increased metabolic demand. This rapid mobilization of energy is particularly important in situations that necessitate heightened cardiac output [19].

Accumulating evidence underscores the role of EAT as a dynamic endocrine organ, distinguished by its ability to generate a unique secretome. EAT synthesizes a diverse array of substances, including vasoactive factors, growth factors, cytokines, and adipokines. These bioactive molecules exert significant biological actions, manifesting either as paracrine effects on myocardial tissues and coronary arteries or through vasocrine mechanisms via the circulatory system [14]. Under physiological conditions, the prominent adipokines produced by EAT are vasoprotective, facilitating vasodilation and demonstrating anti-inflammatory, anti-fibrotic, and antioxidant properties. This multifaceted role of EAT is vital in maintaining cardiovascular homeostasis, suggesting that its dysfunction may contribute to the pathogenesis of various cardiovascular diseases [20].

## 4. The Interplay Between Epicardial Adipose Tissue and Atherosclerosis Development

The relationship between inflammation and coronary artery disease (CAD) has garnered significant attention in recent years, with compelling evidence supporting the connection throughout various stages of the disease. Inflammation is closely linked to all stages of CAD, from the initial development of atherosclerosis to the advancement of atherosclerotic lesions, ultimately leading to plaque rupture and atherothrombosis [21]. The validity of this assertion is further reinforced by the outcomes of the CANTOS trial. This study revealed that the blockade of the interleukin (IL)-1β signaling pathway, through the action of the monoclonal antibody canakinumab, resulted in a considerable decrease in the rates of cardiovascular events. Notably, this reduction occurred independently of any changes in lipid levels [22]. The findings from the CANTOS trial provide crucial evidence supporting the hypothesis that inflammation plays a significant role in cardiovascular disease beyond traditional risk factors associated with lipid levels.

Traditionally, the classical “inside-out” theory posits that atherosclerosis is initiated by injury to intimal endothelial cells, leading to an accumulation of inflammatory cells in the subendothelial space [23]. However, emerging research has introduced the “outside-to-inside” theory, which suggests that inflammation originating in EAT can initiate and propagate atherosclerotic processes inward to the vascular structures [20]. This paradigm shift indicates that stressors such as excessive energy supply, insulin resistance, and diabetes may incite phenotypic alterations in EAT, including hypertrophy and an inflammatory response marked by elevated lipolysis and the release of free fatty acids [24].

Moreover, the inflammatory secretome associated with dysfunctional EAT shifts towards an increased production of pro-inflammatory adipokines such as leptin, resistin, and IL-8. These molecules promote monocyte migration and transition into macrophages, which further exacerbate inflammation through additional cytokine release [25]. The interaction between adipose tissue and the cardiovascular system is thus bidirectional, with vascular signals influencing adipose tissue biology and vice versa [26].

PCAT, situated adjacent to the coronary arterial wall, could play a more localized and direct role in atherogenesis. Its anatomical proximity allows for the activation of inflammatory pathways via paracrine and vasocrine mechanisms, potentially leading to endothelial dysfunction characterized by reduced nitric oxide production, increased hypercoagulability, and vascular smooth muscle cell proliferation [27]. Biopsy studies have confirmed the presence of inflammation in PCAT among patients with advanced CAD, demonstrating a notable upregulation of inflammatory markers in EAT compared to subcutaneous adipose tissue samples [28].

Additionally, non-invasive imaging techniques such as 18F-FDG (fluorodeoxyglucose) positron emission tomography (PET)/CT have illustrated the presence of PCAT inflammation. It is known that the degree of glucose uptake correlates with metabolic activity, particularly in activated inflammatory cells, which typically exhibit increased glucose transporter expression [29]. A case–control study involving non-diabetic CAD patients showed that PCAT had a significantly higher standardized uptake value compared to both other adipose tissue depots and control subjects [30]. However, the clinical application of PET is hindered by limitations such as low spatial resolution and increased radiation exposure. In contrast, CT imaging modalities offer a more accessible means of non-invasively assessing EAT and PCAT inflammation, furthering our understanding of their roles in CAD progression [31]. Thus, the recognition of inflammation as a vital component in atherosclerosis offers promising avenues for therapeutic intervention and diagnostic evaluation.

## 5. Non-Invasive Imaging

### 5.1. Echocardiographic Evaluation of EAT

Transthoracic echocardiography (TTE) has emerged as a vital and efficient tool for visualizing and measuring EAT. This technique is characterized by its simplicity, affordability, and widespread availability, which makes it an ideal choice for clinicians seeking to evaluate EAT in a variety of patient populations. EAT is typically identified as the inhomogeneous hypoechoic space located between the outer myocardial wall and the visceral layer of the pericardium [32].

**Figure 1:**
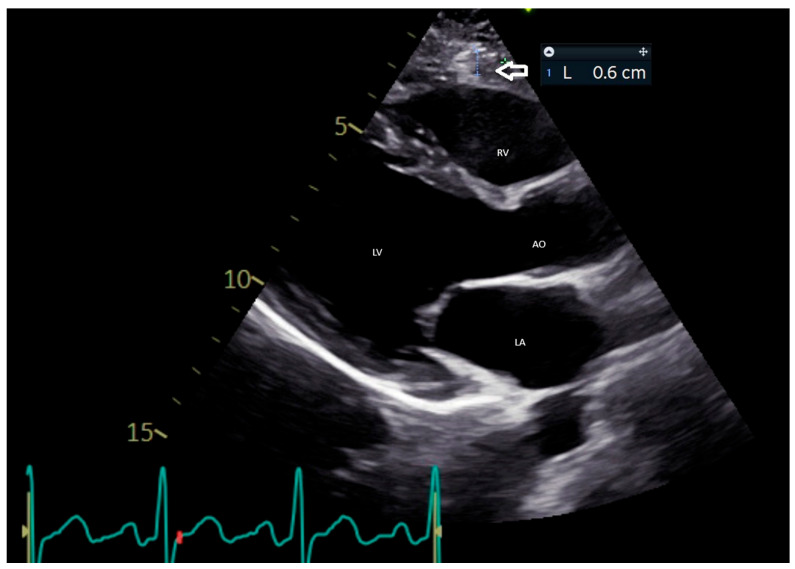
Echocardiographic parasternal long-axis view showing measurement of epicardial fat thickness. AO: aortic root; LA: left atrium; LV: left ventricle; RV: right ventricle.

In clinical practice, EAT thickness is most commonly measured using parasternal long-axis views (Figure 1). This measurement is performed perpendicularly along the free wall of the right ventricle at the end-systolic phase, with values averaged over three cardiac cycles [33]. Iacobellis et al. found that the median thickness of epicardial fat was 6.5 mm in women and 7 mm in men among a cohort of 246 patients who underwent TTE for standard clinical indications [34]. Furthermore, Natale et al. established an upper normal threshold for EAT thickness at 7 mm, derived from findings obtained in a study comprising 50 healthy volunteers [35]. Echocardiographic assessments of EAT thickness have demonstrated a substantial correlation with both the occurrence and severity of coronary artery disease (CAD), as well as the identification of high-risk coronary plaques [36].

Despite the reproducibility of EAT thickness measurements via echocardiography, there are inherent limitations to relying solely on two-dimensional imaging techniques. Such methods do not permit volumetric assessment of EAT, which is crucial for understanding the variability in EAT thickness and calculating total EAT volume. Evidence suggests that a single measurement taken along the right ventricular free wall does not correlate effectively with computed tomography-derived EAT volume [37].

Moreover, the efficacy of echocardiographic imaging is significantly influenced by the individual patient’s acoustic window. In obese patients, where fat impedance can be higher, this can lead to suboptimal imaging conditions [38].

### 5.2. CMR Evaluation of EAT

CMR imaging has emerged as a pivotal tool for assessing total body fat, particularly EAT [39,40], which is increasingly recognized for its role in cardiovascular health. CMR is regarded as the reference modality for imaging total body fat, offering several advantages over other imaging techniques. CMR provides exceptional visualization of both visceral and parietal pericardium, enabling easy assessment and volumetric quantification of EAT, which is crucial for understanding its implications for heart disease [5].

A significant advantage of CMR is its non-invasive nature, as it does not involve radiation exposure or the use of contrast agents. This feature enhances the safety of the procedure, making it a preferred choice for patients who may have contraindications to other imaging modalities. Despite the benefits, CMR has relatively limited clinical availability and is often associated with high operational costs. Additionally, the closed nature of the imaging device can be a barrier for claustrophobic patients or those with implanted devices incompatible with magnetic imaging [41].

**Figure 2:**
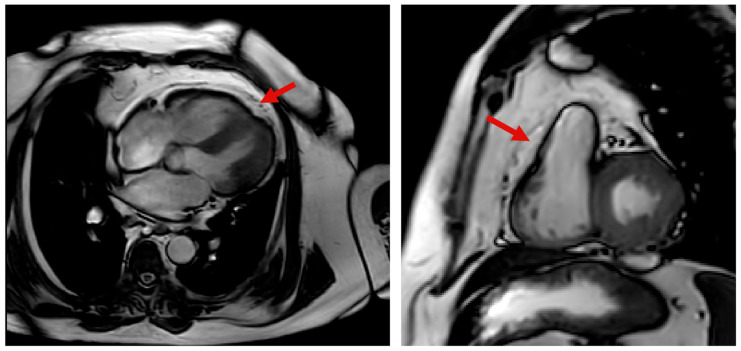
Epicardial adipose tissue visualization (red arrow) at cardiac magnetic resonance in four-chamber (**left**) and short-axis (**right**) steady-state free precession cine sequences.

Various CMR sequences are employed to visualize and quantify EAT effectively. Research indicates that Fast Spin Echo T1-weighted black blood sequences can serve as a reliable method for assessing EAT volume [42]. However, this sequence is not routinely performed in patients referred for CMR, possibly due to technical constraints or the need for specialized expertise. On the other hand, steady-state free precession (SSFP) images (Figure 2), which are traditionally used for evaluating ventricular function and volumes, have proved to be an accurate and reproducible approach for assessing pericardial adiposity [41]. Findings from these sequences reinforce the utility of CMR in quantifying EAT effectively.

The methodology for determining the volume of EAT through CMR entails the manual contouring of the EAT region during end-diastole in short-axis slices, followed by multiplying the EAT area by the slice thickness. The total EAT volume is then obtained by summing the data from all slices [43]. Notably, advancements in imaging technology have led to the development of fully automated quantification techniques for EAT from non-contrast CMR images, further streamlining the process and enhancing accuracy [44].

In conclusion, CMR stands out as a gold standard for imaging EAT due to its advantages in safety, visualization, and quantification. While challenges such as availability and cost persist, ongoing advancements in imaging technology may address these issues and expand the clinical utility of CMR in cardiovascular diagnostics [45].

### 5.3. CCT Evaluation of EAT

Cardiac computed tomography (CT) has emerged as a valuable modality for assessing EAT due to its high spatial resolution and three-dimensional visualization capabilities [46]. Unlike traditional imaging techniques such as echocardiography or CMR, cardiac CT provides detailed insights into the anatomical and pathological features of the heart [47]. This advanced imaging technique allows for more accurate assessment of coronary arteries and a deeper understanding of the relationship between EAT and CAD.

Cardiac CT is routinely employed for coronary artery calcium scoring in asymptomatic individuals, effectively serving as a risk stratification tool [48]. Conversely, coronary CT angiography (CCTA) has become the first-line imaging modality for diagnosing CAD, known for its ability to deliver an excellent prognostic assessment with comparatively low radiation exposure [49] and excellent prognostic assessment [50]. This dual capability to visualize both the coronary artery structure and its immediate environment, particularly PCAT, underscores the significance of CCTA in clinical cardiovascular assessment.

Furthermore, CCTA extends beyond merely evaluating stenosis; it encompasses the characterization of atherosclerotic plaques, identifying high-risk features that may predispose patients to adverse cardiovascular events [39,51]. This includes not only the quantitative measurements of stenosis but also qualitative analyses that highlight the nature and stability of plaques within the arterial walls [52]. Hence, the comprehensive assessment afforded by CCTA proves invaluable in the context of preventive cardiology [39]. However, it is crucial to acknowledge the limitations of the CCTA, particularly concerning exposure to ionizing radiation and the use of iodinated contrast agents. Despite this, significant advancements have been made in reducing radiation exposure while maintaining diagnostic efficacy [49]. This progress renders CCTA a favorable option for patients, balancing diagnostic precision with safety considerations.

In non-contrast computed tomography (NCCT), EAT has attenuation values that span from −190 to −30 Hounsfield Units. Fat voxels located within the visceral pericardium are categorized as EAT, whereas those found within the inner thoracic cavity are designated as thoracic fat [53]. The semi-automated volumetric assessment of EAT involves an expert initially delineating the pericardial contours at intervals of 5 to 10 slices, starting from the bifurcation of the pulmonary trunk and extending to the cardiac apex [37]. Subsequently, software calculates the EAT volume by aggregating the areas from all images, taking into account slice thickness and any gaps between intersections. Mancio et al. conducted a meta-analysis that unveiled an independent relationship between EAT volume and factors such as myocardial ischemia, coronary artery stenosis, and major adverse cardiovascular events (MACEs) [54]. Similarly, Nerlekar et al. highlighted that an increase in EAT volume is linked with high-risk plaque characteristics [55]. Notably, the relationship between EAT and high-risk plaques appears to be more pronounced when assessed through three-dimensional volumetric analysis, as opposed to traditional linear thickness measurements, reinforcing the advantage of volumetric imaging techniques such as CMR and CCT) over echocardiography in EAT evaluation [56].

Moreover, a key threshold of EAT volume between 113 and 120 cm^3^ has been identified as having significant predictive value for future cardiovascular events [57]. While NCCT also allows for the quantification of EAT attenuation, the available data linking this measure to MACE remains contentious. Mahabadi et al. reported that patients who experienced myocardial infarction displayed both higher EAT attenuation and volume [58]. Furthermore, Raggi et al. evidenced that moderate to intensive statin therapy can lead to reduced EAT attenuation, independent of serum lipid levels [59].

Given the time-intensive nature of conventional EAT measurement via semi-automated methods, various software solutions have been developed to streamline this process [60]. A fully automated algorithm utilizing deep learning and convolutional neural networks was introduced for EAT volume and attenuation quantification. This algorithm, applied to cardiac CT scans intended for coronary artery calcium (CAC) scoring, demonstrated an impressive correlation (r = 0.97, *p* < 0.001) with manual measurements [61]. Further analysis revealed that the EAT attenuation and volume quantified using this deep learning approach had substantial predictive value for MACE, independent of the CAC score [57]. Thus, leveraging advanced computational techniques holds promise for enhancing EAT assessment and its implications in cardiovascular health.

### 5.4. PCAT Assessment Through CT

**Figure 3:**
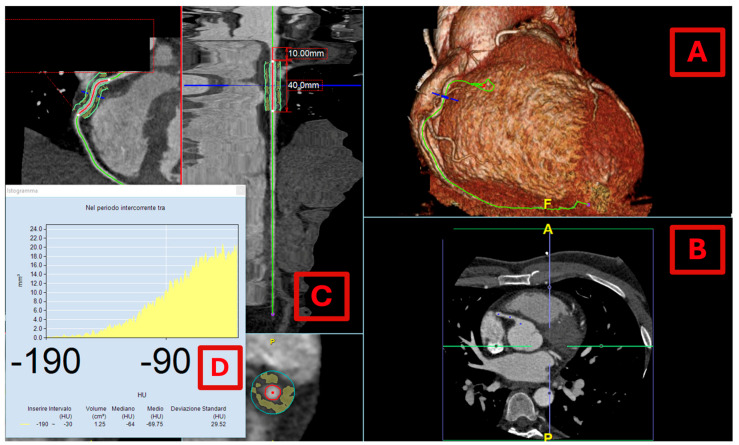
Pericoronary fat attenuation index (pFAI) measurement: Example of how to measure the pericoronary adipose tissue in a male patient with ACS using Aquarius Workstation version 4.4.13.P4 (TeraRecon Inc., Foster City, CA, USA). (**A**) Three-dimensional (3D) coronary CT reconstruction focused on right coronary artery. (**B**) Selection of the right coronary artery (RCA) and its region of interest. (**C**) Pericoronary tissue analysis is performed starting 10 mm from the ostium then the proximal 40 mm segment of the RCA is traced. The adipose tissue within a radial distance from the outer vessel wall equal to the diameter of the vessel is defined perivascular fat. (**D**) Histogram showing the distribution of pericoronary tissue values in the range of −190 and −30 HU. In Table 1, the main advantages and disadvantages of imaging methods are presented.

CCTA allows the meticulous delineation of the vessel wall while simultaneously allowing for the analysis of PCAT [62] (Figure 3), an important factor in understanding the pathogenesis of CAD. Research has highlighted that PCAT volume tends to be elevated around culprit lesions in cases of myocardial infarction and in plaques exhibiting characteristics of vulnerability [63,64].

The interaction between vascular inflammation and PCAT morphology is particularly significant. In states of vascular inflammation, pro-inflammatory cytokines infiltrate the surrounding PCAT, leading to the suppression of pre-adipocyte differentiation. Consequently, this process results in the formation of smaller adipocytes with lower lipid content and higher aqueous content. Furthermore, extracellular edema may contribute to an increase in fluid content within PCAT [65]. These morphological alterations in PCAT result in increased attenuation, which can be quantified by measuring the mean attenuation in voxels containing adipose tissue [66].

A seminal study by Antonopoulous et al. established that normal PCAT, which consists of large, mature, lipid-rich adipocytes, exhibits lower attenuation values (approximately −190 HU). In contrast, inflammation induces the formation of smaller preadipocytes with reduced intracellular lipid, leading to increased PCAT attenuation (approaching −30 HU) [9]. The authors introduced a novel biomarker, the “fat attenuation index (FAI)”, defined as the mean PCAT attenuation within a radial distance from the outer coronary artery wall equivalent to the average vessel diameter [9]. The right coronary artery (RCA) was specifically chosen for analysis due to its lack of significant side branches and the abundant perivascular adipose tissue surrounding it [9]. FAI measurements, taken between 10 and 50 mm from the RCA ostium, revealed that patients with CAD exhibited higher FAI scores compared to those without CAD, with the index correlating with stenosis greater than 50% in any coronary artery [9].

While initial studies focused on the RCA, subsequent investigations demonstrated that PCAT attenuation surrounding the left anterior descending and left circumflex arteries also held prognostic value at the patient level [67]. Goeller et al. further illustrated that in patients presenting with acute coronary syndrome, PCAT attenuation was notably increased around culprit lesions compared to both non-culprit lesions and the highest-grade stenosis lesions in matched stable CAD patients [67,68]. Additionally, Kwecinski et al. explored the relationship between increased PCAT attenuation and high-risk plaque, identifying a correlation with 18F–NaF PET uptake, a promising biomarker for active coronary plaque microcalcification [68].

Additionally, PCAT attenuation around the proximal RCA can successfully distinguish between different stages of CAD and is a predictive marker for the advancement of coronary plaque. A rise in non-calcified plaque burden during a median length of 3.4 years was linked to increased PCAT attenuation, while a decrease in non-calcified plaque burden was linked to lower PCAT attenuation, according to a study comprising 111 stable patients having sequential CTCA [69]. PCAT attenuation around the proximal RCA was significantly higher in patients with acute myocardial infarction than in those with stable CAD and controls without CAD, regardless of total atheroma burden, according to a prospective research by Lin et al. [70].

Unlike static measures such as coronary artery calcium scoring (CAC), which reflects irreversible changes, FAI is dynamic, responding to acute inflammatory events and therapeutic interventions [71,72]. The CRISP-CT study [73], which analyzed data from 3912 patients, demonstrated that elevated perivascular FAI was a strong predictor of cardiac mortality and MACE, independently of traditional risk factors and plaque burden. Furthermore, CRISP-CT showed that FAI values decrease following the initiation of statin therapy, highlighting its potential as a monitoring tool for therapeutic response. The role of PCAT extends beyond risk assessment to therapeutic guidance, as it offers a window into the inflammatory activity driving atherosclerosis progression. Unlike traditional imaging markers that focus on plaque burden and stenosis severity, FAI provides a functional, assessment of disease activity, allowing for a more personalized approach to cardiovascular prevention and treatment [72].

In recent years, a growing body of research has sought to compare intravascular imaging techniques—particularly OCT and IVUS—with CCTA in the evaluation of plaque vulnerability and coronary inflammation. These comparative studies have significantly expanded our understanding of how non-invasive imaging can complement and, in some cases, approximate the diagnostic depth of invasive modalities, especially when enhanced by advanced metrics such as PCAT attenuation, FAI, and radiomic analysis.

The study by Yuki et al. [74] represents one of the most comprehensive efforts to correlate CCTA findings with OCT-defined plaque vulnerability in a large cohort of 474 patients undergoing both imaging modalities. Patients with high PCAT attenuation on CCTA showed a significantly greater prevalence of vulnerable plaque features on OCT—including lipid-rich cores, macrophage infiltration, PR, and microchannels—with the strongest associations observed when PCAT was measured around the culprit vessel. This study firmly established PCAT attenuation as a robust, non-invasive marker of localized coronary inflammation, reinforcing its value in identifying patients at higher risk for ACS. Importantly, the study also demonstrated that this association was markedly stronger in patients with ACS compared to those with stable angina, suggesting that PCAT may capture dynamic, event-related inflammatory processes. Similarly, the radiomics study by Kim et al. [75] took a novel approach by leveraging machine learning algorithms to extract and analyze over 1300 radiomic features from PCAT regions surrounding coronary lesions identified on CCTA. These features—based on shape, texture, and intensity—were then correlated with OCT findings in a smaller but technically rigorous cohort. The results were compelling: radiomic signatures from PCAT were able to predict the presence of TCFA and microchannels with high accuracy, traditionally thought to be detectable only through high-resolution OCT. Another important angle is explored in the study by Lin et al. [76], which examined the relationship between CCs—a well-established marker of plaque vulnerability and inflammation—and PCAT attenuation. Using both OCT and CCTA, the authors demonstrated that plaques containing CCs had significantly higher PCAT attenuation values compared to plaques without. The study also showed that this association held across multiple measurement regions, including lesion-specific, segmental, and proximal reference areas. Notably, PCAT attenuation correlated with the number of CCs, and it decreased after statin therapy, paralleling LDL-C reduction. However, unlike other studies, PCAT was not significantly associated with TCFA or macrophage infiltration, suggesting that PCAT attenuation may selectively reflect crystal-driven inflammation, rather than overall plaque instability.

With the advent of PCAT imaging, FAI, and radiomic analysis, CCTA now offers unprecedented insight into the inflammatory milieu surrounding coronary arteries, bridging the gap between structure and function. These tools now enable us to move from the concept of a vulnerable plaque to that of the vulnerable patient—one with diffuse, active, and inflamed atherosclerotic disease. This integrated, multimodal imaging approach offers the potential for earlier detection, more refined risk stratification, and truly personalized cardiovascular prevention.

## 6. EAT and Treatment Strategies

The primary strategy for addressing EAT involves enhancing physical activity, modifying detrimental behaviors (such as smoking cessation and reducing alcohol consumption), and minimizing caloric intake. Exercise not only diminishes EAT but also ameliorates insulin resistance and mitigates the risk of cardiovascular events [77]. A secondary analysis of a randomized clinical trial, which included 50 patients with abdominal obesity, revealed that 12 weeks of endurance and resistance training led to a significant reduction in EAT mass by 32% and 24%, respectively, in comparison to the control group [78]. Additionally, a decrease in EAT associated with a high-fat and high-protein diet indicated that supplementation with ω-3 polyunsaturated fatty acids (PUFAs) markedly lowered plasma insulin concentrations and the Homeostatic Model Assessment of Insulin Resistance (HOMA-IR) index, thereby reducing EAT [79]. Moreover, left ventricular mass (LVM) and diastolic dysfunction degree exhibited strong correlations with changes in epicardial fat. It was furthermore established that substantial weight loss is correlated with a significant decrease in epicardial fat thickness in severely obese individuals, which serves as a marker of visceral obesity in this population [80].

Regarding pharmacological intervention, drugs that promote the formation of brown adipose tissue enhance energy expenditure, which may ameliorate insulin resistance and lower the incidence of cardiovascular events. Notably, metformin has been shown to enhance insulin sensitivity. In a three-month monotherapy study involving 40 diabetic patients, metformin significantly reduced EAT thickness and body mass index (BMI) [81]. A prospective observational study assessed patients referred for coronary artery bypass grafting (CABG) following acute myocardial infarction, categorizing participants into prediabetic and normoglycemic groups [82]. Within the prediabetic cohort, those who had either not previously used metformin or had undergone nearly six months of metformin treatment prior to CABG exhibited a decrease in EAT volume [82]. Moreover, a randomized controlled trial with 95 patients suffering from type 2 diabetes revealed that the addition of liraglutide notably decreased EAT content [83]. The combination of pioglitazone and simvastatin has been demonstrated to lower levels of pro-inflammatory cytokines IL-6 and TNF-α, thereby diminishing the inflammatory response within EAT and reducing its volume [84]. Glucagon-like peptide-1 receptor agonists (GLP-1 RAs) were identified as effective in enhancing insulin sensitivity, promoting weight loss, EAT mass reduction, increasing adiponectin levels, augmenting insulin secretion, and inhibiting glucagon release, all in a glucose concentration-dependent manner [85]. Another observational study involving 26 type 2 diabetes patients compared a metformin-only group with a group receiving sitagliptin, which was found to significantly reduce EAT [86]. Furthermore, sodium-dependent glucose transporter 2 inhibitors (SGLT-2is) have shown efficacy in curbing sodium-glucose reabsorption in renal proximal tubules, with both dapagliflozin and empagliflozin significantly decreasing EAT mass [87]. The meta-analysis by Myasoedova et al. demonstrated that GLP-1 RA and SGLT2-i—as well as statins—significantly reduce EAT. Among these, GLP-1 RA had the most pronounced effect, followed by SGLT2-i, while statins showed a mild benefit [88]. The therapeutic response was notably stronger in younger individuals with higher BMI, suggesting these drugs may be particularly effective in high-risk patients [88]. These findings position EAT not only as a biomarker but also as a modifiable therapeutic target in cardiometabolic disease management.

## 7. Conclusions

When adversely remodeled and dysfunctional, epicardial adipose tissue (EAT) releases pro-inflammatory adipokines that significantly contribute to CAD development. PCAT, which surrounds the coronary arteries, exhibits a distinct phenotype compared to non-PCAT EAT, emphasizing its importance in cardiovascular health. Noninvasive imaging techniques provide valuable tools for quantifying EAT, with cardiac CT emerging as the most effective method. Notably, CCTA enables a comprehensive assessment, facilitating the simultaneous evaluation of coronary artery stenosis, plaque characteristics, and PCAT attenuation. This attenuation metric is gaining recognition as an independent biomarker of coronary inflammation, yet its clinical integration requires careful consideration of certain limitations. The current understanding of PCAT attenuation’s natural progression and its response to standard treatments in patients with stable CAD or following acute coronary syndromes remains incomplete. Furthermore, standardizing PCAT attenuation measurements across various CT systems and scanning protocols is essential before its routine clinical application. Future research is warranted to validate PCAT’s utility in risk assessment, ultimately aiding in the customization of targeted CAD therapies and enhancing primary and secondary prevention strategies.

## Figures and Tables

**Table 1:** Imaging methods for EAT evaluation.

Imaging Methods	Measurements	Advantages	Disadvantages
Echocardiography	Thickness	- Low cost; - No ionizing radiation; - Noninvasive; - Widely available; - Frequently performed for other reasons.	- Low reproducibility between and within operators; - Lacks volumetric measurement; - Restricted to right ventricular free wall; - Inferior image quality caused by limited acoustic windows, particularly in obese individuals.
Cardiac Magnetic Resonance	Thickness Area Volume	- No ionizing radiation; - No iodinated contrast; - Volumetric measurement; - Multiparametric and multiplanar imaging; - Adipose tissue imaging gold standard.	- High cost; - Restricted accessibility; - Extended scanning duration and decreased comfort; - Unsuitable for individuals with severe obesity; - Constraints on bore magnet size for those who are severely obese.
Cardiac Computed Tomography	Thickness Area Volume	- Consistency between and within readers; - Volumetric measurement; - PCAT assessment; - Coronary Artery assessment; - Short scanning duration.	- High cost; - Ionizing radiation exposure; - Iodinated contrast use; - Weight capacity limit for individuals with severe obesity.

Abbreviations: EAT—epicardial adipose tissue; PCAT—pericoronary adipose tissue.

## Abbreviations

ASCVD	atherosclerotic cardiovascular disease
BMI	body mass index;
CABG	coronary artery bypass grafting;
CAD	coronary artery disease;
CCTA	cardiac computed tomography angiography;
CMR	cardiac magnetic resonance;
CT	computed tomography;
EAT	epicardial adipose tissue;
FAI	fat attenuation index;
GLP-1 RA	glucagon-like peptide-1 receptor agonist;
NCCT	non-contrast computed tomography;
PAT	pericardial adipose tissue;
PCAT	pericoronary adipose tissue;
PET	positron emission tomography;
PVAT	perivascular adipose tissue;
RCA	right coronary artery;
SGLT-2i	sodium-dependent glucose transporter 2 inhibitor;
TTE	transthoracic echocardiography.
